# Deficient vergence prism adaptation in subjects with decompensated heterophoria

**DOI:** 10.1371/journal.pone.0211039

**Published:** 2019-01-18

**Authors:** Anna Przekoracka-Krawczyk, Krzysztof Piotr Michalak, Paulina Pyżalska

**Affiliations:** 1 Laboratory of Vision Science and Optometry, Faculty of Physics, Adam Mickiewicz University, Poznań, Poland; 2 Vision and Neuroscience Laboratory, NanoBioMedical Centre, Adam Mickiewicz University, Poznań, Poland; University of Muenster, GERMANY

## Abstract

Vergence prism adaptability was evaluated in subjects with high symptomatic and asymptomatic heterophoria and compared to individuals with a heterophoria within normal range (the control group). A computer haploscope was used to measure phoria values and changes in the eye position after introducing 6 prism diopters base out in front of the right eye. Phoria values were measured with a nonius paradigm every minute for a period of 10 minutes during adaptation. The results showed that subjects with symptomatic heterophoria are characterized by a lower rate of prism adaptation and adapted to a smaller extent with respect to the control group. The group with high but asymptomatic heterophoria showed prolonged adaptation time but after several minutes of binocular viewing the subjects were able to adapt to the prism to a level similar to the control group. These findings suggest that an impairment in the slow vergence control system may be responsible for the inability to fully reduce vergence effort, which results in poor vergence ranges and asthenopic symptoms during prolonged viewing.

## Introduction

During natural viewing the two eyes are directed very precisely to a target of regard and the image of the target object is projected close to the center of the retina in each eye. In a nonstrabismic individual, the two images can be fused in the brain if they are of adequate quality. However, under specific clinical and laboratory test conditions the target may be presented to only one eye to prevent fusion so that an angular deviation of the non-stimulated eye may occur. This deviation is referred to as heterophoria or phoria [1]. Even small amounts of phoria may lead to visual symptoms (asthenopia) unless they are compensated by the vergence system. Inversely, even significant phoria may remain asymptomatic if the individual level of compensatory skills is sufficient [2–4]. Thus, it seems that compensatory and adaptation processes are needed to improve functional efficiency and accuracy of the visual system. One of the adaptation processes related to heterophoria is vergence prism adaptation (VPA) also known as phoria adaptation. As a result of VPA induced phoria returns to baseline (tonic) value [5, 6]. This can be the case after several minutes of sustained viewing of a near object or when looking through a prism, which increases vergence effort. This shift in the amount of phoria is probably a consequence of a changed tonic position of the eyes [7] and is a useful mechanism to reduce near visual stress during prolonged viewing [1]. This adaptation mechanism may be activated in response to disparity evoked using a stereoscope or prism spectacles as well as in response to accommodation through lenses [8–11].

Krishnan & Stark’s hypothesis [12] and a later model proposed by Schor [5, 13] states that two neural components are responsible for vergence control, i.e. fast and slow vergence controllers. When disparities between the retinal images from the two eyes are detected (for example evoked by a prism), the fast vergence system rotates the eyes to achieve fusion. However, prolonged viewing through a prism and/or at near causes excessive vergence effort. Thus, after several minutes of sustained binocular fixation the slow vergence system changes the vergence state back to the baseline-tonic phoria [5].

The role of the slow fusional component is to reduce the output of the fast fusional vergence response and to decrease the effort of the fast fusional vergence mechanism [6, 14]. It is believed that VPA reflects the response of the slow vergence component. The model of fast and slow components of vergence control was described in a neurological study where two types of vergence-related cells in the midbrain were described. These hypothetical burst neurons were supposed to correspond to the fast vergence controller and tonic neurons—to the slow vergence system [15]. According to this model, any defect in the slow fusional mechanism may evoke an excessive demand from the fast system causing asthenopic visual symptoms [16]. In other words, it is not the heterophoria per se that causes visual stress (asthenopic symptoms) but it is attributable to an impaired adaptation ability of the slow vergence system. Despite the fact that the "fast" burst neurons have not been found empirically, studies on subjects with binocular vision deficits and heterophoria support this hypothesis. For example, Ogle et al. [17] reported impaired VPA, especially at distance, where symptomatic heterophoria was present. North and Henson [18] found that the majority of the examined patients with symptomatic heterophoria demonstrated lack of/deficient prism adaptation. Carter [19] noted that subjects with high heterophoria but proper VPA skills did not exhibit visual symptoms. Similar observation made by North and Henson [18] suggests that subjects with poor adaptation ability report asthenopic problems. Carter [19] claimed that VPA should be taken into account when anticipating visual symptoms. These findings were also confirmed by a more recent study conducted by Sreenivasan and Bobier [20] who observed reduced vergence adaptation skills in subjects with convergence insufficiency, including cases induced by traumatic brain injury. Additionally, Brautaset & Jennings [21] demonstrated that symptomatic subjects with decompensated phoria showed a deficient VPA mechanism both in the convergent and divergent direction both at near and distance. Similarly, reduced VPA at near and distance in response to base-in and base-out prisms was found by Nilsson and Brautaset [22]. All the described subjects exhibited excessive convergence. Improvements in the slow vergence component’s performance have been reported also in patients with convergence insufficiency following orthoptic treatment. [23–25]. The authors concluded that an increase in positive fusional vergence results from changes in the speed and magnitude of VPA and that orthoptic treatment leads to an increase in convergence peak velocity and a decrease of convergence accommodation.

VPA is a phenomenon of great importance for the oculomotor system although mechanisms underlying this process remain not fully understood. Investigation of the correlation between VPA and asthenopic symptoms in subjects with abnormal binocular vision seems necessary since it could help specialists to better understand the nature of binocular deficits and allow them to determine treatment plans. The aim of our study is to verify a hypothesis claiming that impaired neural mechanism underlying VPA, not heterophoria per se, may be a major cause of asthenopic symptoms. We have investigated VPA in subjects with high decompensated (symptomatic) heterophoria (dCPh) compared to individuals with high compensated (asymptomatic) heterophoria (CPh) and in subjects with phoria within normal range (NPh), to determine how the process of vergence adaptation differs between these groups. We found that subjects from the dCPh adapted the prism to a smaller extent with respect to the NPh and CPh, showing that visual symptoms might be determined by the poor VPA but not by the high phoria per se.

## Materials and methods

### Subjects

A group of forty five subjects took part in the experiment. The participants were students of Adam Mickiewicz University in Poznań, Poland and patients of the Optometry Centre, Adam Mickiewicz University Foundation in Poznań. The students received extra points toward their course grades as compensation for their participation. All subjects gave their informed consent prior to entering the study. Each participant was screened for visual acuity and refractive error. In order to assess binocular skills, cover test was administered to measure heterophoria at near and at distance, 4-dot Worth test was used to detect any suppression or diplopia, Titmus test was applied to measure stereopsis, pencil push-up method was used to determine the near point of convergence and Risley prisms were used to get vergence ranges at far and near. Additionally, the COVD (*College of Optometrists in Vision Development*) questionnaire was administered which assesses the incidence of 19 visual symptoms in 5 degrees of severity: “never”, “seldom”, “sometimes”, “often” and “always” (each answer is respectively 0, 1, 2, 3 or 4 points). A total score of 20 or more suggests the presence of potential visual problems. According to this, patients with more than 20 points were classified as symptomatic [26]. All refractive errors were corrected and all subjects wore appropriate prescribed lenses during all the tests administered as part of the main experiment.

Inclusion criteria for all study subjects were: good corrected visual acuity (at least 0.0 logMAR) for both eyes, fusion without diplopia, stereopsis at least 50 seconds of arc, no manifest strabismus, no history of strabismus and no vertical phoria. Depending on their visual results, the subjects were divided into three groups:

1. **dCPh group** (decompensated—symptomatic phoria)–subjects with (a) decompensated heterophoria higher than 2 Δ eso or 4 Δ exo at distance and 2 Δ eso or 7 Δ exo at near, (b) narrow vergence ranges (i.e. failed Sheard’s criterion—fusional reserves lower than double the value of phoria [27]) and (c) visual symptoms (a score higher than 20 points in the COVD questionnaire). This group consisted of 15 subjects, age ranged from 18 to 38 years (mean = 26), 9 females and 6 males. Detailed visual parameters are listed in Table 1.
2. **CPh group** (compensated—asymptomatic phoria)–subjects with (a) compensated heterophoria higher than 2 Δ eso or 4 Δ exo at distance and 2 Δ eso or 7 Δ exo at near but (b) experiencing no visual symptoms (less than 20 points in the COVD questionnaire) due to wide vergence ranges and fulfilled Sheard’s criterion (fusional reserves higher than double the value of phoria [27]) and (c) no interocular suppression and normal stereopsis. There were 15 subjects in this group as well, age ranged from 22 to 32 years (mean = 25), 6 females and 9 males. Detailed visual parameters are listed in the Table 1.
3. **NPh group** (phoria within normal range)–subjects with no heterophoria or phoria within normal range [27] who reported no visual symptoms (less than 20 points in the COVD questionnaire) and wide vergence ranges. No interocular suppression and normal stereopsis were confirmed. This group also consisted of 15 subjects, age ranged from 22 to 39 years (mean = 29), 10 females and 5 males. Detailed visual parameters are listed in the Table 1.

**Table 1:** Visual parameters of the subjects.

	BCVA OD [logMAR]	BCVA OS [logMAR]	Far phoria [Δ]	Near phoria [Δ]	Fusional reserves far [Δ]	Fusional reserves near [Δ]	Stereopsis [sec of arc]	COVD [score]	Number of subjects with esophoria *far/near*	Number of subjects with exophoria *far/near*
	*mean* (SD)	*mean* (SD)	*mean median* (SD)	*mean median* (SD)	*mean median* (SD)	*mean median* (SD)	*mean* (SD)	*Mean* (SD)		
**NPh**	-0.07 (0.08)	-0.07 (0.07)	0.5; 0.0; (0.8)	1.2; 0.0; (1.8)	7.9; 8.0; (1.0)	14.6; 14.0; (2.1)	40 (0.0)	15 (2.3)	2/0	2/6
**CPh**	-0.07 (0.07)	-0.08 (0.08)	7.5; 7.0; (3.5)	10.0; 9.0; (3.6)	15,2; 14,0; (7.4)	26.6; 26.0; (6.8)	41 (2.6)	17 (1.3)	4/4	11/11
**dCPh**	-0.06 (0.07)	-0.06 (0.07)	8.7; 8.0; (3.5)	10.1; 10.0; (2.8)	6.8; 7.0; (3.7)	11.2; 12.0; (3.5)	41 (3.5)	29 (3.8)	6/6	9/9

The results of phoria measurements using cover test are presented as absolute values. Fusional reserves in subjects without heterophoria are given as the lower value of blur (base in or base out) found during vergence ranges measurements. OD—right eye, OS—left eye, BCVA—best corrected visual acuity, SD—standard deviation, Δ—prism diopters, NPh—normal range phoria group, CPh—compensated phoria group, dCPh—decompensated phoria group.

The study was approved by the local Ethics Committee at Adam Mickiewicz University and was performed in accordance with the Declaration of Helsinki. Subjects gave written consent to participate in the study and were informed that they could discontinue their participation at any stage of the experiment.

### Apparatus

**Fig 1:**
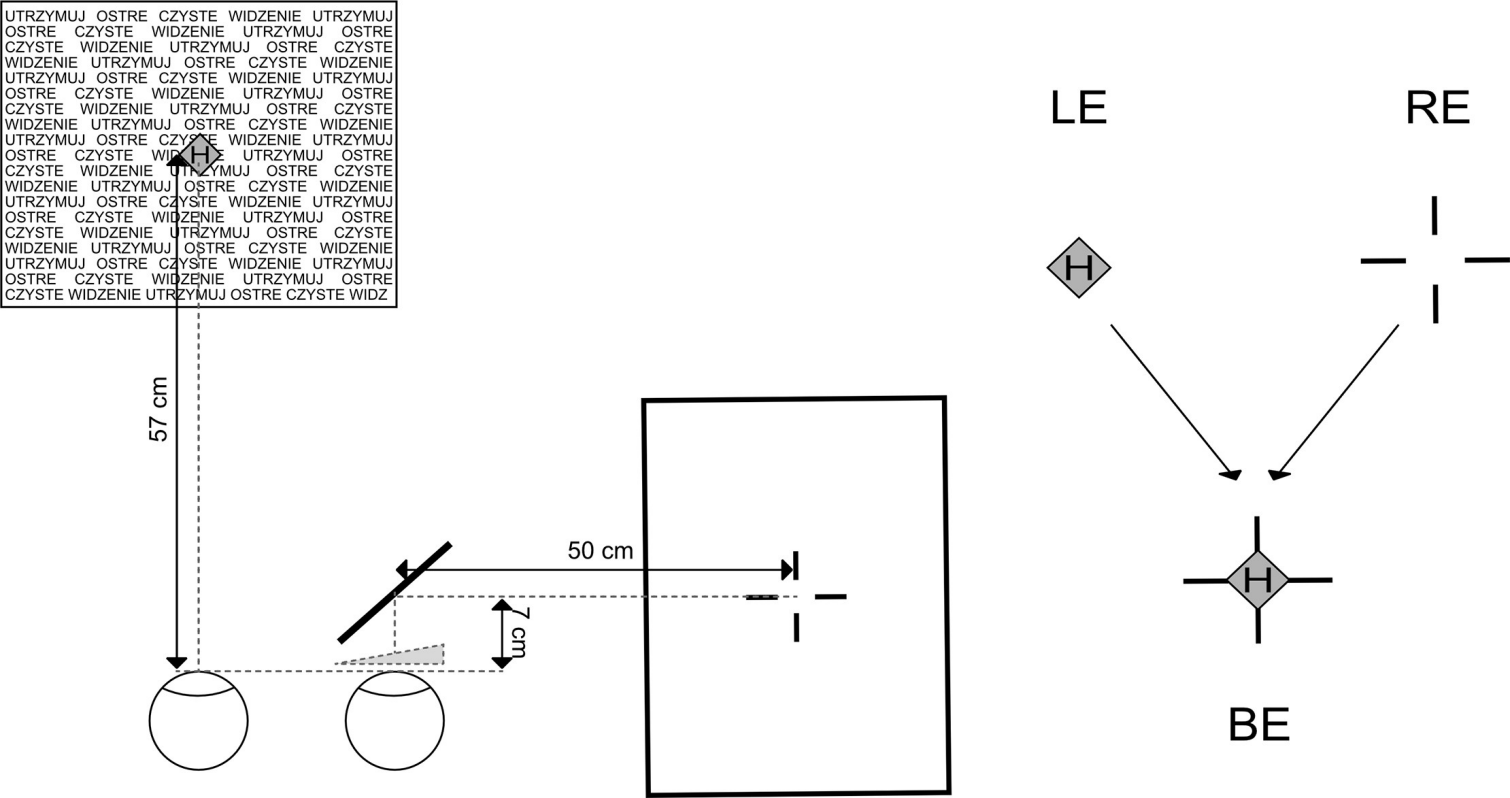
Computer haploscope. M1 –monitor 1, M2 –monitor 2, M–a mirror tilted 45 deg of arc with respect to the vertical fronto-parallel plane, P–prismatic googles with 6 Δ base out prism in front of the right eye and a plano lens in front the left eye. On the right: letter H on a green diamond (seen by the left eye) and a red cross (seen by the right eye) which was centered with letter H during phoria measurements.

Measurements were taken using a computer haploscope with design similar to the one used by Kono et al. [28]. The device (see Fig 1) consisted of two 22-in. LCD monitors (monitor 1 and monitor 2) positioned perpendicularly to each other at 57 cm from the subject's eyes. A headrest and a chinrest were used to stabilize the subject’s head and eliminate any vestibular influences. A folding mirror (size 12 cm x 8 cm) tilted 45 deg of arc with respect to a vertical fronto-parallel plane was placed in front of the right eye. The mirror enabled the subjects to see an image displayed on the monitor which was perpendicular to the subject’s face (monitor 1) but obstructed the field of view of the left eye in order to prevent fusion.

Special goggles with a 6 Δ base out prism in front of the right eye and a plano lens in front of the left eye were used. The goggles induced approximately 6 Δ of exophoria. If the subject required prescription, the goggles were placed on top of their spectacles.

### Stimuli and procedure

The experiment consisted of three parts: Part 1 (Pre-test)–where phoria was measured using a computer haploscope without the prismatic goggles. The measurement was taken 3 times and the results were averaged. The averaged value was considered subject’s baseline phoria. The baseline phoria was recorded by computer and was subtracted from the phoria measured during Part 2 (the adaptation phase). Part 2—during the adaptation phase, subjects were allowed to adapt to 6 Δ base out prism for 10 minutes. In this part, phoria measurements were taken at regular intervals of one minute with the subjects wearing prismatic goggles. Part 3 (Post-test)—where phoria was measured 3 times and averaged just after the prismatic goggles have been removed.

During phoria measurements, subjects were instructed to look with their left eye on the letter H placed in a background of green diamonds (size 0.69 deg of arc) surrounded by matrix letters (stimulus for accommodation—matrix size: 20.3 x 15.6 deg of arc, single letter: size 0.3 x 0.2 deg of arc). The right eye, looking into the mirror, saw a red cross (size: 2.1 deg of arc) (see Fig 1). The participants moved the cross to the center of the letter H using computer keyboard arrows. The subjects were required to move the cross very fast and confirm its position by pressing the space bar on the keyboard. The position of the cross was recorded by the software. Prior to each phoria measurement, the right eye was occluded for 15 seconds to ensure phoria stabilization [13, 28]. During the prism adaptation phase, the mirror was removed and a column of letters (total size: 19.8 x 0.2 deg of arc) was presented in front of both eyes to keep binocular fixation and fusion. The subjects looked at the column of letters through prism googles for one minute between each phoria measurement. This procedure was repeated 10 times (a total 10 minutes of adaptation).

Henson and North [29] showed that VPA is maximal around 3 minutes of binocular viewing and Brautaset and Jennings [30] found that VPA was complete by 5 minutes to a 6 prism base-out, so they chose usually 7 minutes of adaptation in their studies [21, 31, 32]. To be sure that VPA will be full or almost full, 10 minutes of adaptation was chosen in the current study.

The experiment was controlled and data was saved using Presentation Software (ver. 15.1, Neurobehavioral Systems, Inc.).

### Analysis

In order to assess the adaptation level (final phoria) and the rate of phoria adaptation, data for each individual subject was fitted to an exponential decay function with the following formula (1)

$$y=a+b*e^{-t/c}$$ (1)

where *a* is the final value that the exponential decay function approaches, *b* is the magnitude of adaptation required from the first measurement trial to reach value *a*, *t* is the time of adaptation (one minute), *c* (the decay constant) is the rate of which the adaptation takes place [33–35].

In the current study all the subjects used goggles with a 6 Δ base out prism in front of the right eye, which induced 6 Δ of exophoria (which has a negative value). Therefore, function (1) was modified as follows (2)

$$y=a+(-a-6)*e^{-t/c}$$ (2)

The parameters of the fitted function provided information concerning the ability of prism adaptation for each subject individually. In this model, parameter *a* was taken as the final phoria (expressed in prism diopters) i.e., that is the amount of phoria induced by the prism which remained not adapted after 10 minutes of adaptation. The smaller the value of the parameter *a*, the greater the ability to reduce the phoria induced by the prism. When the *a* parameter is large, it means that the person is poorly adapted to the prism. In order to verify how many prism diopters of induced phoria the subject could adapt to, it is necessary to subtract the *a* value from the 6 Δ (which is the full amount of phoria induced by the prism). The exponential decay constant (*c)* is the time (expressed in minutes) required for prism adaptation to obtain 63.2% (1-*e*^-1^) of the final level of adaptation, which means about 2/3 of the final phoria [36]. The higher the value of the parameter *c*, the faster the subject reduces the phoria induced by the prism.

Additionally, the *aftereffect* was calculated, which was the value of phoria remaining after the prism goggles were removed. The *aftereffect* was a difference in phoria values between the Post- and Pre-test phase. Its direction should be opposite to the direction of the shift caused by the prism during an adaptation phase. A high value of the aftereffect means that the subject reached a high level of adaptation.

All curve fits were generated using Wolfram Mathematica Software (ver. 8.0.1, Wolfram Research, Inc.). Two subjects from the CPh group and four from the dCPh group showed no VPA and their data could not be fitted to the function. In those cases, for statistical analysis, mean phoria from the last three minutes of adaptation was assumed to be equal to the value of parameter *a*, and the value of 6 (~60% of the adaptation time) was used as parameter *c*.

Statistical analysis was conducted using Statistica Software (ver. 10, Stastoft, Inc.). Shapiro-Wilk test was used to verify normal distribution of the data. Data (parameter *a*, *c* and *aftereffect*) had normal distribution thus were analyzed using parametric tests: ANOVA and Tukey’s posthoc test to compare results between the groups.

Additionally, it was checked whether the phoria after removing the prism in the Post-test (Part 3) had been changed significantly from the baseline value (in the Pre-test—Part 1). For this purpose, the *aftereffect* was compared with zero value for each group separately, using one-sample t-test. If the *aftereffect* was significantly higher than zero, then the baseline phoria had changed as a result of adaptation—the higher the *aftereffect*, the more prism adaptation occurred. However, if the *aftereffect* was not significantly higher than zero, then no prism adaptation took place.

The phoria values measured with the cover test was also compared statistically to see whether the NPh differed significantly from the CPh and the dCPh groups. The data had abnormal distribution, thus nonparametric Mann-Whitney U test was used.

The differences were considered statistically significant when *p* was equal or less than 0.05.

## Results

First of all, statistical analysis showed that absolute phoria at distance and near for the CPh and the dCPh groups was significantly higher than for the NPh group (distance phoria: Z = -4.78, p < 0.001 for CPh and NPh, respectively and Z = -4.77, p < 0.001 for dCPh and NPh, respectively; near phoria: Z = -4.72, p < 0.001 for CPh and NPh, respectively and Z = -4.64, p < 0.001 for dCPh and NPh, respectively). However there were no significant differences in phoria values at distance or at near between the CPh and the dCPh groups (distance phoria: Z = -1.05, p = 0.295; near phoria: Z = -0.79, p = 0.426) (see Table 1).

### Prism adaptation

**Fig 2:**
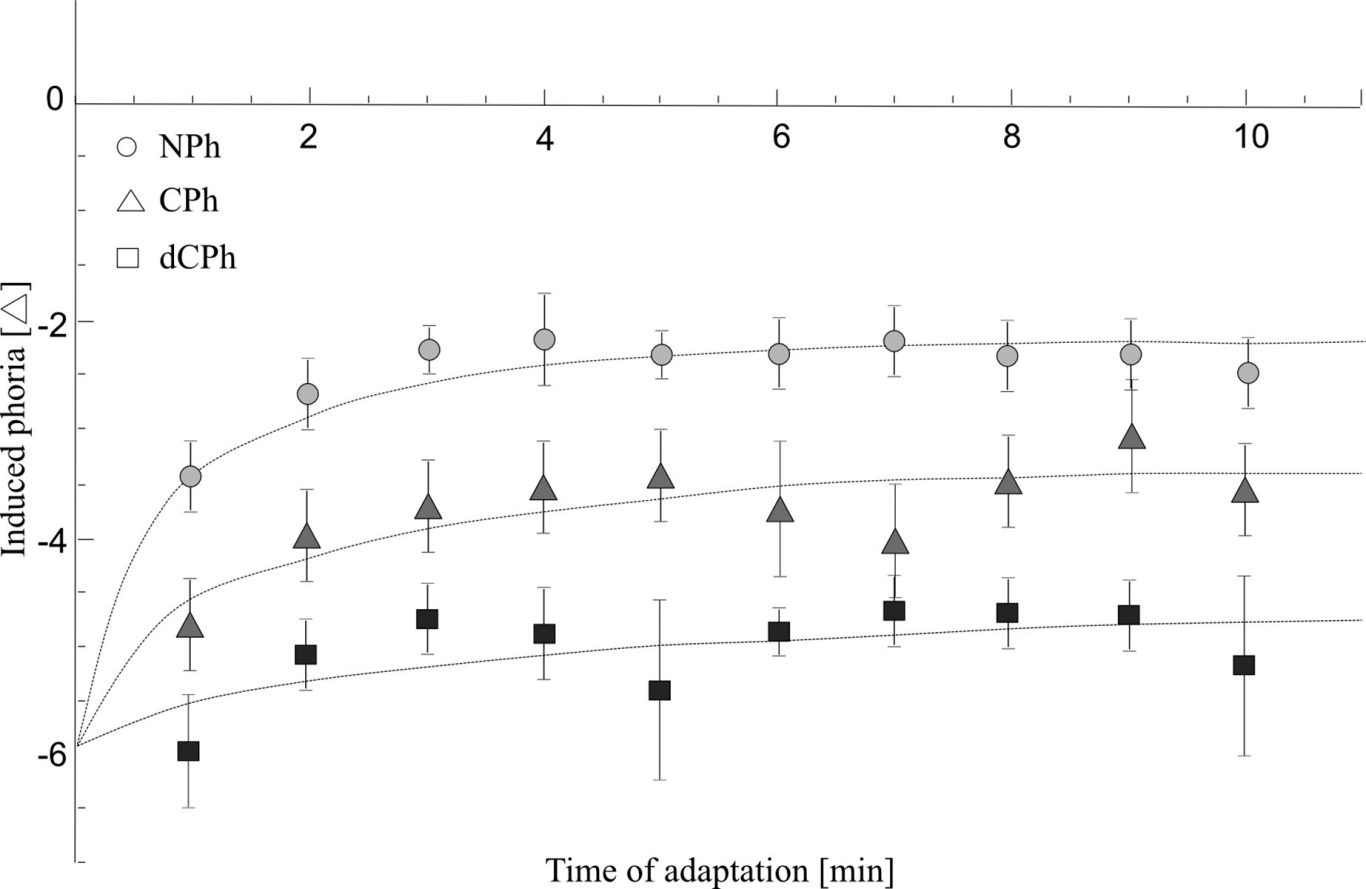
Vergence prismatic adaptation. Vertical axis shows the level of induced phoria and the horizontal axis indicates the time taken to reach adaptation. NPh—normal range phoria group, CPh—compensated phoria group, dCPh—decompensated phoria group.

**Fig 3:**
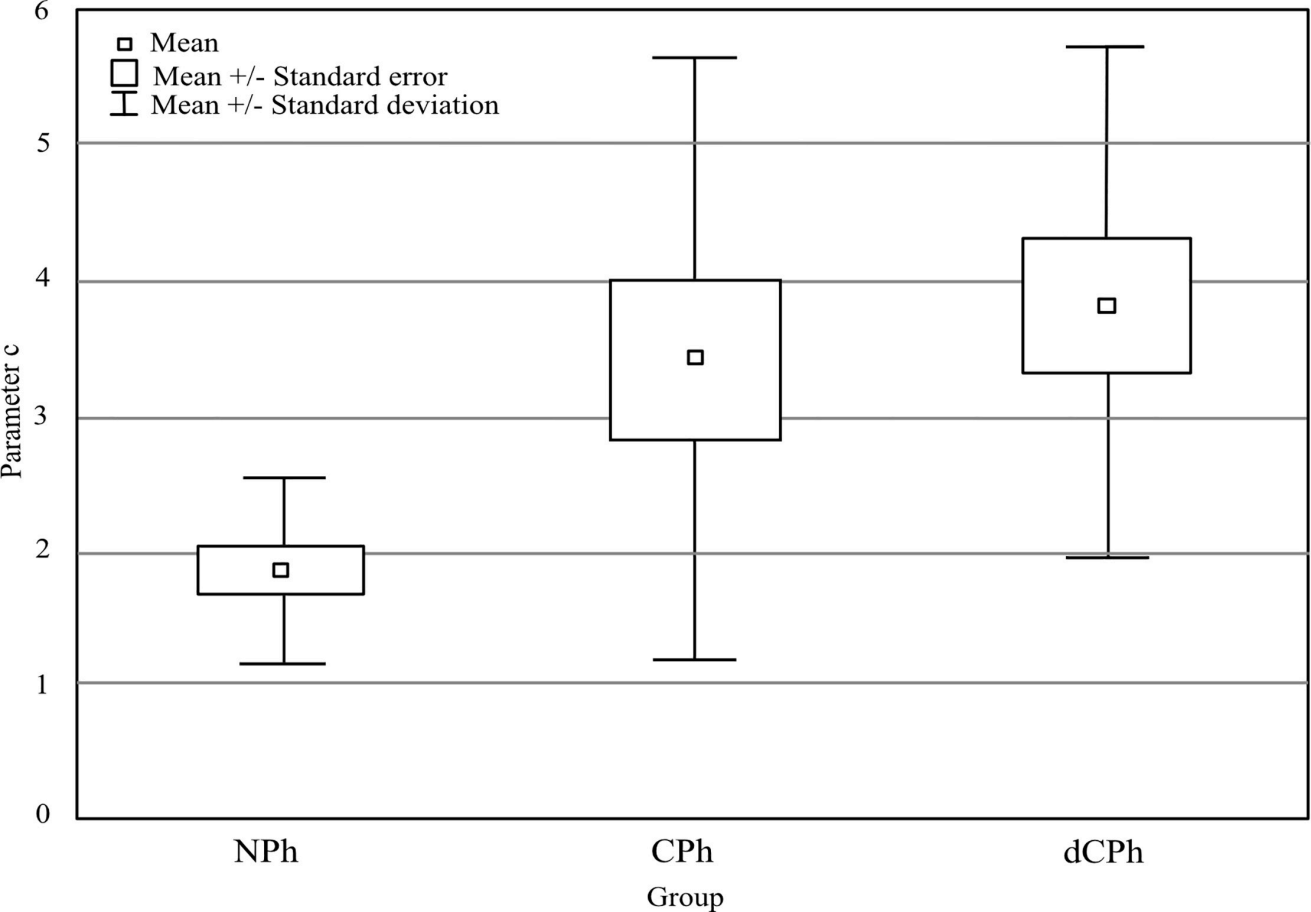
Adaptation rate–parameter *c*. NPh—normal range phoria group, CPh—compensated phoria group, dCPh—decompensated phoria group. The small squares represent an average value of phoria; the large squares represent the standard error and the vertical bars mark the standard deviation.

Fig 2 presents changes in phoria value during 10 minutes of adaptation, and Fig 3 shows value of parameter *c*. As can be seen there, subjects from the NPh group adapted to prism at the highest rate, as compared to the other groups. The difference in parameter *c* between the groups was indicated by a significant main affect of group *(*F(2, 42) = 5.42, p = 0.008, η^2^ = 0.21). Posthoc test showed that the CPh and the dCPh groups needed almost 4 minutes to adapt 2/3 of the final level of adaptation (parameter *c*: 3.4 vs 3.8, for the CPh and the dCPh group) and the difference in parameter *c* between the groups was not statistically significant (p = 0.779). NPh group adapted to prism much faster, reaching 2/3 of the final level of adaptation in only 1.9 minutes (significant difference between the CPh and the NPh, p = 0.047; significant difference between the dCPh and the NPh, p = 0.009).

**Fig 4:**
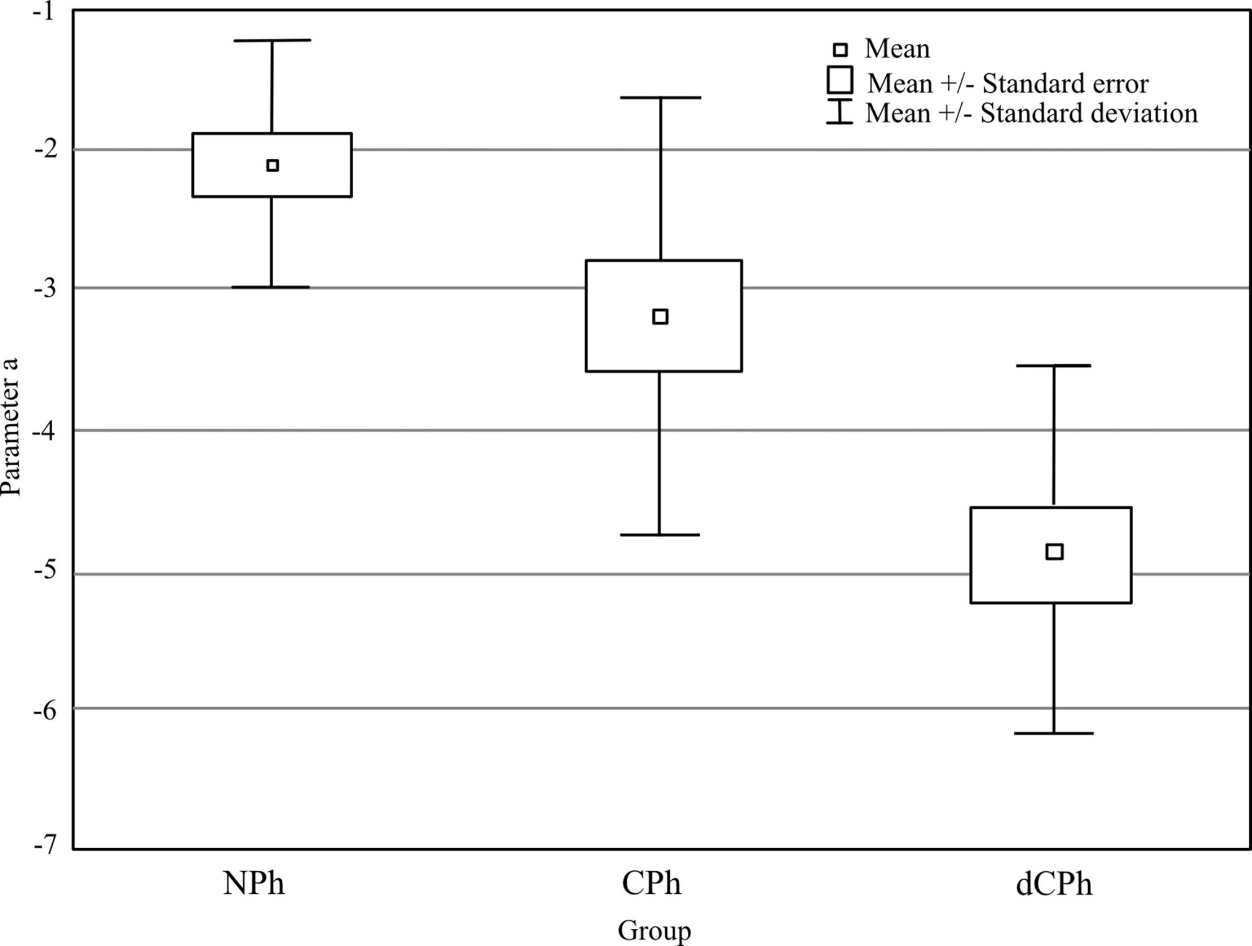
Final level of adaptation–parameter *a*. NPh—normal range phoria group, CPh—compensated phoria group, dCPh—decompensated phoria group. The small squares represent an average value of phoria; the large squares represent the standard error and the vertical bars mark the standard deviation.

Value of parameter *a* is presented in Fig 4. As can be see, differences in VPA were observed also with regard to parameter *a* (main effect of group: F(2,42) = 17.51, p < 0.001, η^2^ = 0.45). As could be expected, ability to prism adaptation of the dCPh group was very weak (parameter *a*: -4.9) comparing both to the NPh (parameter *a*: -2.1, posthoc p < 0.001) and to the CPh group (parameter *a*: -3.2, p = 0.003). When value of *a* has been subtracted from 6 (phoria induced by the prism) it was clear that after 10 minutes of adaptation the dCPh adapted only 18% (6–4.9 = 1.1 Δ) of induced phoria.

Despite the fact that the CPh group adapted to prism slower than the NPh, which was indicated by parameter *c*, they were able to adapt to a similar level as the NPh (parameter *a*: -2.1 vs -3.2, for the NPh and the CPh, posthoc p = 0.066) and better than the dCPh group (parameter *a*: -3.2 vs -4.9, for the CPh and the dCPh, posthoc p = 0.003). When value of *a* has been subtracted from 6 (phoria induced by the prism) it could be seen that after 10 minutes of adaptation the CPh group adapted 47% (6–3.2 = 2.8 Δ) and NPh 65% (6–2.1 = 3.9 Δ) of induced phoria.

### The aftereffect

**Fig 5:**
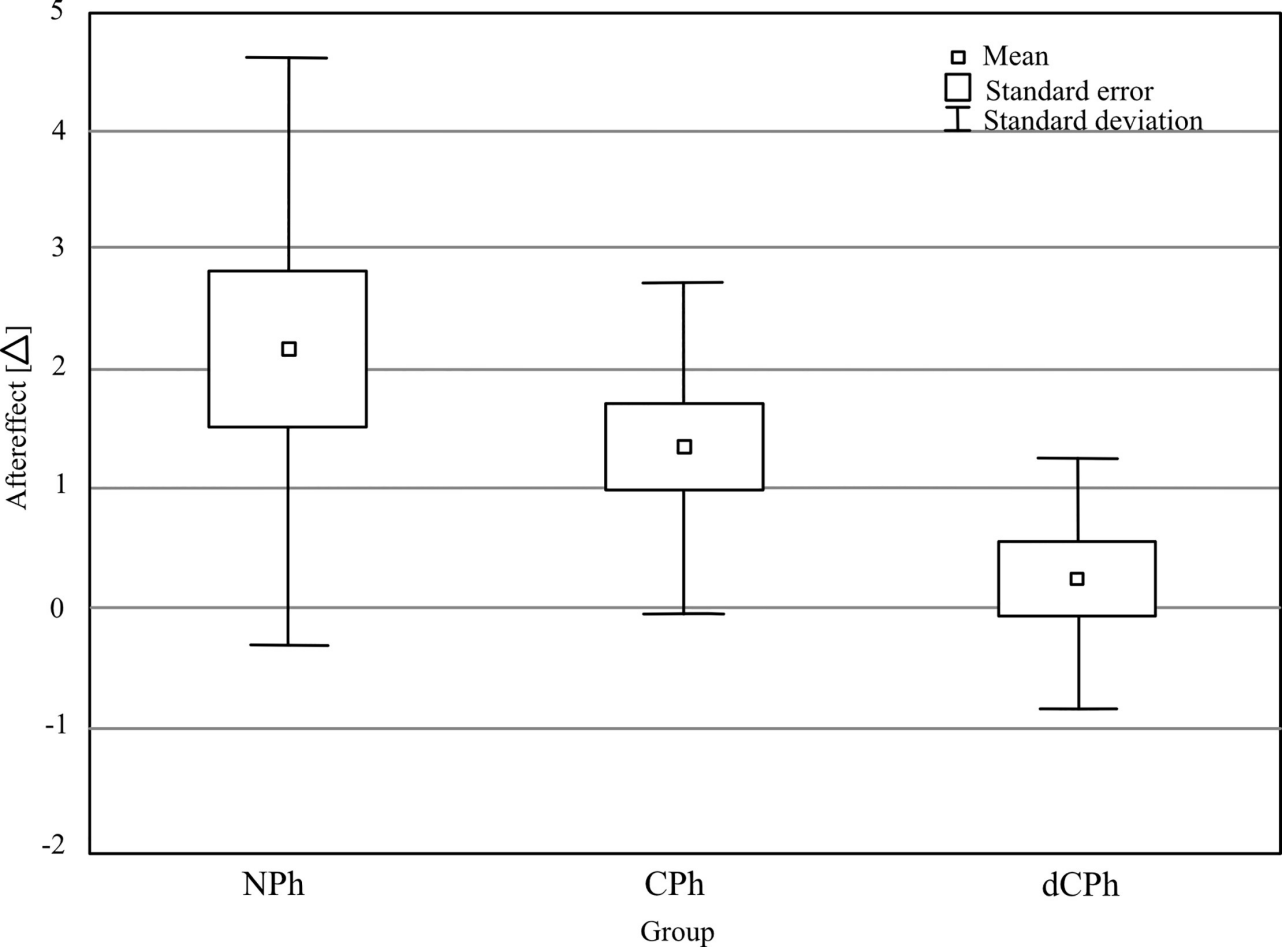
Aftereffect. NPh—normal range phoria group, CPh—compensated phoria group, dCPh—decompensated phoria group. The small squares represent an average value of phoria; the large squares represent the standard error and the vertical bars mark the standard deviation.

The value of aftereffect is shown in Fig 5. As can be observed, phoria measured after 10 minutes of adaptation when prism googles were removed, as expected, had the opposite direction to the inducing prisms (*aftereffect*). The aftereffect was the highest in the NPh group (+2.2 Δ), lower in the CPh group (+1.4 Δ) and the lowest in the dCPh group (+0.22 Δ) (main effect of group: F(2,42) = 4.95, p = 0.012, η^2^ = 0.19). The posthoc test indicated that the difference in the *aftereffect* was significant only between the NPh and the dCPh group (p = 0.009). Additionally, Student t-test demonstrated that induced phoria after removing the prism was significantly higher than zero for both the NPh (t(14) = 3.41, p = 0.004) and the CPh groups (t(14) = 3.81, p = 0.002) but not for the dCPh (t(14) = 0.76, p = 0.461).

## Discussion

The present study focused on the investigation of VPA skills in symptomatic and asymptomatic individuals with heterophoria. We tried to answer the question whether impaired VPA may be related to asthenopic symptoms independent of the amount of phoria.

Two groups of subjects with high heterophoria (CPh and dCPh) were selected to the study because they could have deficits in vergence system and VPA. Their results were compared with subjects without any binocular vision disorder and with efficient vergence system (NPh). The results indicated that subjects from the dCPh group showed deficient VPA reflected by slow adaptation rate when compare to the NPh and low amount of adaptation when compared to both the NPh and CPh groups. They needed almost 4 minutes to adapt 2/3 of the final phoria and after 10 minutes of binocular viewing they adapted only 18% of induced phoria. Impaired prism adaptation was reflected also by phoria values measured after removing the prism *(aftereffect)* as only marginal phoria in the opposite direction to the induced prism was found in this group. These results are in agreement with the findings of other studies, showing deficient prism adaptation in subjects with symptomatic abnormal binocular vision [18–21, 23]. After 10 minutes of binocular viewing, subjects from the NPh group adapted 65% of induced phoria, and they needed less than 2 minutes to adapt 2/3 of the final phoria’s value, which is 43% of the induced demand. The third study group (subjects with compensated phoria—CPh) demonstrated interesting results. They were slower in adaptation than the NPh but after 10 minutes they were able to adapt to prism to a level just below the NPh’s result. The *aftereffect* proved also that the CPh group was able to adapt to a similar level as the NPh. However, lower adaptation rate (parameter *c*) in the CPh, as compared to the NPh group, suggests that certain VPA deficits may still be present in individuals with high heterophoria (even when its compensated). The comparison showed that two aspects of VPA should be considered: the rate of adaptation and the magnitude of adaptation, since they could be two different mechanisms.

One can argue that slow adaptation rate in the CPh group could have resulted from the high amount of phoria that had to be compensated by the vergence system. In case of high phoria, additional prism introduced in front of the eyes might evoke large retinal disparity. Large retinal disparity may require more time to be reduced by a slowly responding vergence system and thus subjects with high phoria may need more time to adapt induced retinal disparity as compared to subjects with orthophoria or low heterophoria. What is important, despite prolonged adaptation time, subjects from the CPh were able to adapt to a similar prism power as the NPh, which was indicated by parameter *a* and the *aftereffect*. It suggests that reduced vergence skills (such as narrow vergence ranges) and asthenopic symptoms occur in subjects who are not able to adapt fully to prism but not necessarily demonstrate slow adaptation rate. This view is supported by the Schor and Ciuffreda hypothesis [37] who claimed that residual retinal disparity, termed fixation disparity (FD), occurred due to inaccuracy of binocular fixation. This, in turn, became an input for the slow vergence system, which is responsible for maintaining vergence posture and relieving stress of the fast controller [13]. The slow vergence controller changes the tonicity of the eye muscles as long as there is any FD. However, in case of binocular disorders, the slow vergence system is not able to maintain a proper vergence posture and reduce FD, causing strain of the fast controller, which leads to visual symptoms during sustained viewing. It suggests that sustained FD, often observed in subjects with decompensated phoria [1, 38, 39] or individuals with reading problems [40, 41], might be treated as an indicator of impaired VPA. FD was not measured in the present study but in future studies it would be interesting to evaluate the correlation between FD and VPA skills in subjects with symptomatic heterophoria, dyslexia and patients with neurological (cerebellar) disorders.

In the latest study of Santos et. al. [42] differences in phoria adaptation to symmetrical and asymmetrical base out prisms were found in subjects with normal binocular vision. The authors suggested that the differences could be even more pronounced in convergence insufficiency (CI) patients because the vergence system in CI patients seems to be impaired. Similar to our study, base out prisms were used in this experiment and it would be interesting to find whether the difference between adaptation to symmetrical and asymmetrical prisms would be more noticeable between symptomatic and asymptomatic patients.

Neurophysiology of VPA has not been fully explained to date but it seems justified to treat it the same way as other oculomotor learning mechanisms. Studies of the cerebellum indicated that this brain structure might play a major role in the VPA process since it is also required for normal oculomotor adaptation during smooth pursuits [43], saccades [44, 45], vestibulo-ocular reflex [43, 46, 47] as well as for response to prisms which shift visual fields laterally [48, 49]. Nitta et al. [50] demonstrated that Purkinje cells modulate their firing rate in response to vergence stimuli and confirmed that these neurons are engaged in motor learning to reduce movement errors [45]. The oculomotor vermis receives signals concerning binocular disparity and vergence-related information from the pons [51]. Therefore, it seems logical that this cerebellar region may be also involved in VPA. Takagi et al. [52] demonstrated that lesions of the dorsal vermis in primates reduced the amount of phoria adaptation. Additionally, Milder and Reinecke [53] examined the ability of prism adaptation in subjects with cerebellar deficits. They confirmed reduced VPA ability which could not be explained by lower fusional reserves or misalignment of the eyes since these parameters were compared to a healthy control group. They claimed that VPA is a cerebellar dependent response. Similar observations were reported later by Hain and Luebke [54]. Also, Kono et al. [28] were interested whether the cerebellum contributes to phoria adaptation. They examined the ability to adapt to vertical prisms in patients with cerebellar disorders. A lower prism adaptation in those subjects was found even when they did not demonstrate manifest strabismus or limited eye movements. It is possible that other structures than the cerebellum could be responsible also for impaired VPA in their subjects since some of them demonstrated additional damages to the pons or the brain stem. However, as suggested in this study [28], no limitation of eye movements in these patients and proper Hering’s law of conjugacy suggest that the ocular mechanisms related to the brain stem were intact. Their patients demonstrated ocular deficits typical for cerebellar lesions [55].

Considering the studies mentioned above, it seems justified to suggest that deficient VPA in decompensated phoria subjects may result from impairments at the level of the cerebellum. It is possible that because of cerebellar disfunction neural integrators for phoria adaptation might fatigue very quickly [28]. This, in turn, could be reflected by low adaptation rate and inability to fully reduce fixation disparity. The idea that the cerebellum may be involved in controlling VPA and other oculomotor deficits was also suggested in other studies which demonstrated impaired ability of implicit motor learning (one of the fundamental cerebellar functions) in subjects with binocular problems [56] and dyslexia [40], poor body balance control in adults [57] and children [58] with strabismus or changes in walking strategies applied by patients with strabismus [59]. All of these functions depend, at least to some extent, on cerebellar control.

To summarize, the present study demonstrated that subjects with decompensated heterophoria suffer from deficient VPA, which might explain poor vergence ranges and asthenopic symptoms during sustained fixation. Individuals with high but asymptomatic heterophoria showed lower adaptation rates but after 4 minutes of binocular viewing they were able to adapt to a similar level as subjects with orthophoria or phoria within normal range. This suggests that the inability to fully adapt to induced fixation disparity may cause stress in the vergence system leading to oculomotor symptoms.

## Supporting information

S1 Dataset (XLS)
